# Protocol based evaluation for feasibility of extubation compared to clinical scoring systems after major oral cancer surgery safely reduces the need for tracheostomy: a retrospective cohort study

**DOI:** 10.1186/s12871-018-0506-8

**Published:** 2018-04-20

**Authors:** Axel Schmutz, Rolf Dieterich, Johannes Kalbhenn, Pit Voss, Torsten Loop, Sebastian Heinrich

**Affiliations:** 1 Department of Anaesthesiology and Critical Care Medicine, Medical Center, University of Freiburg, Faculty of Medicine, Hugstetter Strasse 55, 79106 Freiburg, Germany; 2 Department of Oral and Maxillofacial Surgery & Regional Plastic Surgery, Medical Center, University of Freiburg, Faculty of Medicine, Hugstetter Strasse 55, 79106, Freiburg, Germany

**Keywords:** Primary tracheostomy, Major oral cancer surgery, Difficult airway management, Airway obstruction, Difficult intubation, Difficult extubation

## Abstract

**Background:**

Despite risks, complications and negative impact to quality of life, tracheostomy is widely used to bypass upper airway obstruction after major oral cancer surgery (MOCS). Decision to tracheostomy is frequently based on clinical scoring systems which mainly have not been validated by different cohorts. Delayed extubation in the Intensive Care Unit (ICU) may be a suitable alternative in selected cases. We hypothesize that delayed routine ICU extubation after MOCS instead of scoring system based tracheostomy is safe, feasible and leads to lower tracheostomy rates.

**Methods:**

We retrospectively analyzed our clinical protocol which provides routine extubation of patients after MOCS in the ICU. The primary outcome measure was a composite of early reintubation within 24 h or secondary tracheostomy. Secondary outcome measures included airway obstruction related morbidity and mortality. Predictor variables included tumor localisation, surgical procedure and reconstruction method, length of operation and pre-existing morbidity. Furthermore we assessed the ability of four clinical scoring systems to identify patients requiring secondary tracheostomy. Statistical processing includes basic descriptive statistics, Chi-squared test and multivariate logistic regression analysis.

**Results:**

Two hundred thirty four cases were enclosed to this retrospective study. Fourteen patients (6%) required secondary tracheostomy, Ten patients (4%) required reintubation within 24 h after extubation. No airway obstruction associated mortality, morbidity and cannot intubate cannot ventilate situation was observed. Seventy five percent of the patients were extubated within 17 h after ICU admission. All evaluated scores showed a poor positive predictive value (0.08 to 0.18) with a sensitivity ranged from 0.13 to 0.63 and specificity ranged from 0.5 to 0.93.

**Conclusions:**

Our data demonstrate that common clinical scoring systems fail to prevent tracheostomy in patients after MOCS. Application of scoring systems may lead to a higher number of unnecessary tracheostomies. Delayed routine extubation in the ICU after MOCS seems an appropriate and safe approach to avoid tracheostomy and the related morbidity.

**Electronic supplementary material:**

The online version of this article (10.1186/s12871-018-0506-8) contains supplementary material, which is available to authorized users.

## Background

Tracheostomy is the standard approach to bypass upper airway obstruction after major oral cancer surgery (MOCS) with reconstructive tissue transfer [1, 2]. However the necessity of routine tracheostomy has been questioned due to associated various complications and due to the negative impact on swallowing function and quality of life. Hemorrhage, obstruction, via falsa, local infection, pneumonia, tracheal stenosis and malignoma recurrence due to malignoma seeding have been reported with incidence between 4 to 8% [3–7]. Furthermore the effect of tracheostomy on functional and social rehabilitation and hospitalization is difficult to quantify but may be underestimated [8–10].

Delayed extubation in the ICU may be an alternative to avoid tracheostomy but is related with difficult airway management in these patients. The equipment for difficult airway management like videolaryngoscopic devices, staged extubation wires and staged reintubation catheters as well as the clinical procedures to manage difficult airway situations has shown enormous development in the past few years [11, 12]. Consecutively the spectrum of safely manageable extubations and possible difficult reintubations exceeded.

To identify patients with indicative need for tracheostomy and those patients who can be safely treated without tracheostomy, multiple clinical scoring systems have been published previously [13–16]. However the published clinical scoring systems often lack of evaluating delayed extubation as possible option to avoid tracheostomy [17].

With the goal to avoid tracheostomy and consecutively decrease time to functional recovery, minimize iatrogenic trauma and morbidity, delayed extubation in the ICU is our institutional standard approach after MOCS. We hypothesize that delayed routine extubation in the ICU after MOCS is a safe and feasible approach and leads to a lower incidence of tracheostomies than application of common clinical scoring systems. Therefore we aimed to evaluate common clinical scores regarding their ability to predict the need for tracheostomy in our cohort. Further objective of the study was to develop and evaluate a score to predict secondary tracheostomy or early reintubation in patients of our cohort.

## Methods

**Fig. 1:**
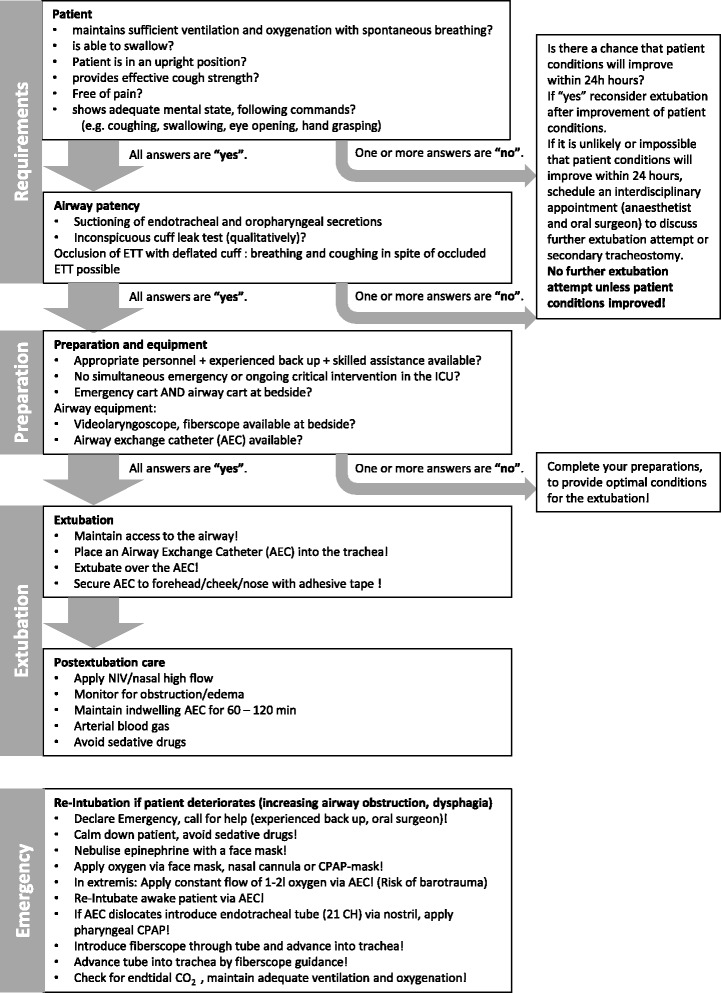
Standard Operating Procedure for high-risk extubation. Extubation of the difficult airway. In accordance with the guidelines published in [27]

This retrospective cohort study was approved by the local Ethics Committee, University of Freiburg, Germany (approval number EK 330/16). The study was conducted at the Department of Anesthesiology and Intensive Care and the Department of Oral and Maxillofacial Surgery & Regional Plastic Surgery, University Medical Center, Freiburg, Germany. The study was conducted according to the Strengthening the Reporting of Observational Studies in Epidemiology (STROBE) Statement: guidelines for reporting observational studies. The STROBE checklist is enclosed to the Additional file 3. The study was initiated in 2016, the retrospective data collection was conducted in 2016. Due to initiation of an electronical patient data and management system in 2012 which allows to gain the relevant data, we enclosed only files of 2012 or later. The study cohort consists of all consecutive MOCS cases between 2012 and 2016. The observational retrospective study design declines the need for a priori sample size calculation. The sample size is the result of enclosing all MOCS patients from 2012 until the end of data collection in November 2016. The standard operating procedure for high-risk extubation is shown in Fig. 1.

**Fig. 2:**
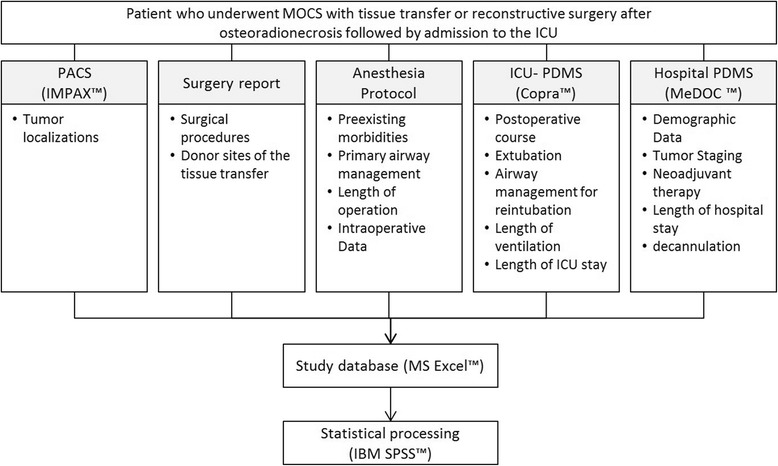
Study protocol, collection and processing of the study data. ICU: intensive care unit | PACS: picture archiving and communication system | PDMS: patient data management system

The closed consecutive cases of patients who underwent MOCS with tissue transfer for reconstruction followed by admission to the ICU between 2012 and 2016 were analysed. Figure 2 shows the protocol of data collection and statistical processing for the study. To avoid selection bias, we cross-checked all cases of postoperative ICU-admissions during the observation period with the tumour register of the Department of Oral and Maxillofacial Surgery & Regional Plastic Surgery. Preoperative classification was made according to preoperative staging CT-Scans and reliable records.

Prior to operating procedures all cases were discussed in the local interdisciplinary tumor board meetings. The case records were reviewed for general demographic data and specific predictor variables like surgical procedure and reconstruction method, length of operation and pre-existing morbidity, malignoma localization [18], neoadjuvant therapies, hospital mortality and discharge information. Surgical information like donor site of the tissue transfer, intraoperative complications (e.g. anastomosis revision), requirement of artificial tissue (Epigard™ Biovision, Ilmenau Germany or Mucograft™ Geistlich, Wolhusen Switzerland) were retrieved form the surgical report. With regard to the airway management we analyzed primary airway management (OR) and if applicable the secondary airway management in the ICU, the need for primary or secondary tracheostomy, the need and timing for reintubation, management of extubation, length of mechanical ventilation and length of ICU stay.

As primary measure a composite outcome was defined by secondary tracheostomy or early reintubation within 24 h after primary extubation. Secondary outcome measures included airway obstruction related morbidity and mortality as well as the incidence of emergency airway punctures. For reintubations more than 24 h after extubation other reasons than airway obstruction were causative. Secondary tracheostomy after reintubation was not mandatory but only performed, when airway obstruction by flap tissue or prolonged mechanical ventilation was expected.

Sedation of the patients was stopped after admission to the ICU. Patients solely received opioids (morphine or piritramid) to achieve tolerance for endotracheal tube. Extubation was only performed when patients were fully awake, able to swallow and after an inconspicuous cuff-leakage test. In cases the attending intensivist expected extubation failure to be likely an airway exchange catheter was placed through the endotracheal tube prior to extubation and remained until airway obstruction was unlikely.

The data was collected in a MS Excel™ (Microsoft, Redmond, USA) datasheet. Further statistical processing was performed using SPSS™ (IBM, Armonk, USA). Statistical testing included basic descriptive statistics for quantitative variables, Chi-squared test and multivariate logistic regression analysis.

Using the patients of our cohort we evaluated the following scores regarding their ability to predict the need for tracheostomy: Kruse Score [15], Gupta Score [14], Kim Score [13], Cameron Score [16]. The detailed parameters of the evaluated scores are shown in Additional file 2.

Predictors of secondary tracheostomy or reintubation within 24 h after extubation were analyzed using Chi squared test. The enclosed parameters of the univariate analysis are shown in Additional file 1. Based on the results of the analysis we developed a study score to assess whether the primary endpoint defined by secondary tracheostomy or early reintubation could be predicted with clinical parameters. Furthermore we validated the parameters of the study score with a multivariate regression analysis.

## Results

**Fig. 3:**
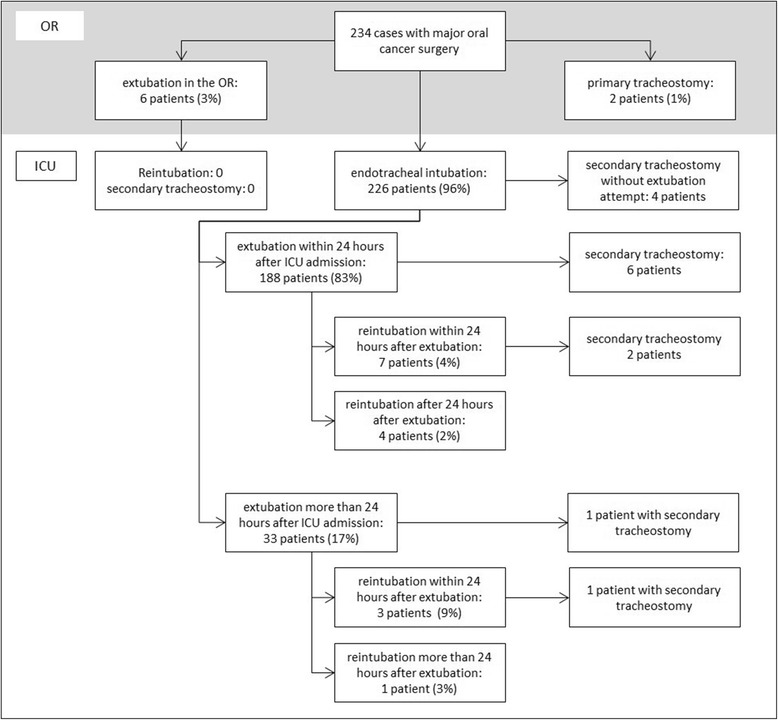
Postoperative course and requirement for airway management

The patients’ characteristics including tumor sites, operative approaches and postoperative courses are shown in Table 1. A total of 234 case records were enclosed to the study. The detailed ICU course of the patients and postoperative requirements for airway management are shown in Fig. 3.

**Table 1:** Patients’ characteristics

Demographic Data	Entire cohort *n* = 234	With sec. Trach. or early reintubation (< 24 h) *n* = 21	Without sec. Trach. or early reintubation (< 24 h) *n* = 213
Age [years]	63 (54 | 73)	64 (60 | 71)	62 (54 | 62)
BMI [kg/m^2^]	24 (21 | 28)	23 (21| 25)	25 (21 | 29)
Male / female	130 (56%) | 104 (44%)	11 (52%) | 10 (48%)	119 (56%) | 94 (44%)
ASA and Mallampati Score			
ASA 1 | 2| 3| 4 | missing ASA	15 (6%) | 110 (47%)| 98 (42%)| 6 (2.6%) | 5 (2.1%)	0 | 10 (48%) | 10 (48%) | 0 |1 (5%)	15 (7%) | 100 (47%) | 88 (41%)| 6 (3%) | 4 (2%)
Mallampati 3&4	65 (28%)	7 (33%)	58 (27%)
Mallampati 1&2	118 (50%)	11 (53%)	107 (50%)
Mallampati missing	51 (22%)	3 (14%)	48 (23%)
Tumor localisation			
hard palate	25 (11%)	2 (10%)	23 (10%)
soft palate	15 (6%)	1 (5%)	14 (6%)
anterior floor of the mouth	19 (8%)	1 (5%)	18 (8%)
anterior floor of the mouth with mandibula	46 (20%)	9 (43%)	37 (16%)
posterior floor of the mouth	5 (2%)	0 (0%)	5 (2%)
posterior floor of the mouth with mandibula	52 (22%)	7 (33%)	45 (19%)
buccal (+ other localization) | solitary buccal	34 (15%) | 9 (4%)	4 (19%) | 1 (5%)	30 (13%) | 8 (3%)
anterior lingual (+other) | solitary anterior lingual	67 (29%) | 34 (15%)	3 (14%) | 0 (0%)	64 (27%) | 34 (15%)
Surgical procedures			
Unilateral neck dissection | bilateral neck dissection	99 (42%) | 61 (26%)	5 (24%) | 7 (33%)	94 (40%) | 54 (23%)
Latissimus dorsi	25 (11%)	10 (48%)	15 (6%)
Radial forearm flap	40 (17%)	4 (19%)	36 (15%)
Reconstruction plate, no primary osseous reconstruction	62 (26%)	6 (29%)	56 (24%)
Non vascularized iliac crest	4 (2%)	1 (5%)	3 (1%)
Microvascular osseous reconstruction	26 (11%)	7 (33%)	19 (8%)
Pelvic	5 (2%)	0 (0%)	5 (3%)
Scapula	10 (4%)	4 (19%)	6 (3%)
Fibula	11 (5%)	3 (14%)	8 (3%)
Minor reconstruction with artificial tissues	39 (17%)	0 (0%)	39 (18%)
Times	Median (25% Quartile | 75% Quartile)		
Length of operation [h]	6.0 (4.0|9.7)	11 (9.5 | 14)	5.8 (3.9 | 8.7)
Length of ICU stay [h]	21.7 (18.7|48.7)	160 (89 | 260)	21.3 (18.4 | 40)
Time from ICU admission to extubation [h]	8.5 (4.1|17.0)	15 (14 | 24)	7.4 (4 | 16.4)
Postoperative airway management and complications (n)			
Primary tracheostomy	2 (1%)	0	2 (1%)
Composite primary measure (Sec. tracheostomy or early reintubation)	21 (9%)	21 (100%)	0
Secondary tracheostomy	14 (6%)	14 (67%)	0
Surgical tracheostomy	10 (71%)	10 (48%)	0
Dilatative tracheostomy	4 (29%)	4 (19%)	0
Reintubation	15 (6.4%)	10 (48%)	5 (2%)
Early reintubation (< 24 h)	10 (4.3%)	10 (48%)	0
Cardiac arrest	3 (1.3%)	0	3 (1.4%)
Hospital Mortality	1 (0.4%)	1 (5%)	0

Categorical variables were given as absolute number and percentage. Continuous variables were given as median (25%quartile | 75%quartile)

We observed two airway related cardiac arrests: bronchial hypersecretion led to a hypoxemia 9 h after primary extubation and upper airway obstruction caused by swelling because of venous flap congestion. In both cases, patients regained return of spontaneous circulation (ROSC) after reintubation. Both patients showed an inconspicuous further clinical course without signs of neurological impairments. Furthermore we observed no ICU or operation related mortality or severe morbidity. A single patient died during the hospital stay three months after the primary operation due to massive postoperative tumor progress and refusal of further therapy.

Secondary tracheostomy was performed as non-emergency procedure in all cases. Emergency surgical airway management such as emergency airway puncture was not observed in our cohort. One patient received primary tracheostomy due to excessive tumor resection; another patient already had a nearly closed tracheostomy prior to the operation which was reopened in the OR after the operation. Both patients were attributed to primary tracheostomy for this study. In 46 cases the ICU physician expected the extubation failure to be likely and an airway exchange catheter was placed through the endotracheal tube prior to extubation. Reintubation was performed by flexible fiberoptic bronchoscopy in 7 cases, by videolaryngoscopy in 4 cases and by conventional direct laryngoscopy in 4 cases. Emergency airway puncture was not required in any of the cases.

Table 2 shows the detailed results of the scores published by Cameron [16], Kim [13], Gupta [14] and Kruse [15]. The suggested number of tracheostomies ranges from 17 (Kruse Score) to 117 (Cameron Score). The positive predictive value ranges from 0.08 (Cameron Score) to 0.18 (Gupta Score). The scores published by Cameron [16], Kim [13] and Gupta [14] showed an explicit attribution of the patients to “need for tracheostomy” or “no need for tracheostomy”. The Kruse [15] score contains of a group of patients with intermediate risk. 25 of our patients met the criteria for intermediate risk. In favor of a better comparability, these patients were attributed to the “no need for tracheostomy” group. An attribution to the “need for tracheostomy” group would have resulted in poorer specificity (0.83) and a slightly increased sensitivity (0.25) of the Kruse score.

**Table 2:** Evaluation of the clinical scores

	Cameron [16]	Kruse^a^ [15]	Kim [13]	Gupta [14]
Suggested tracheostomy n / %	117 / 50%	17 / 7%	23 / 10%	44 / 19%
True positive	10	2	4	8
False negative	6	14	12	8
False positive	107	15	19	36
True negative	111	203	199	182
Sensitivity	0.63	0.13	0.25	0.5
Specificity	0.50	0.93	0.91	0.84
Positive predictive value	0.08	0.12	0.17	0.18
Negative predictive value	0.95	0.94	0.94	0.96

^a^25 Patients with intermediate risk were attributed to the group without the need for tracheostomy

**Fig. 4:**
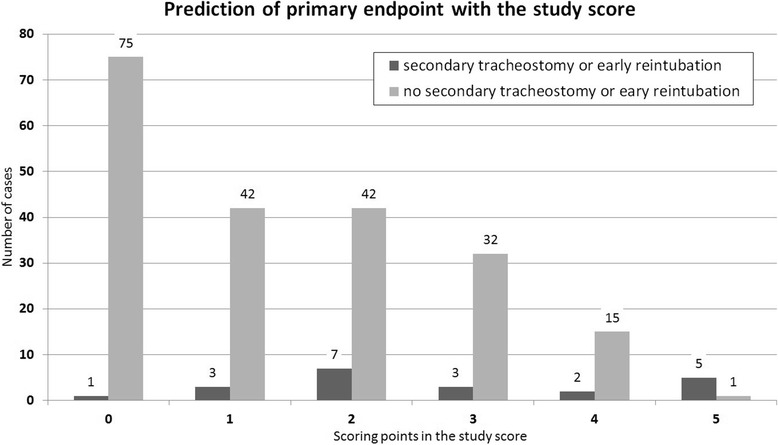
Prediction of secondary tracheostomy or early reintubation with the study score. Items for the study score: Length of operation exceeding 75% quartile, tumor site at pars alveolaris mandibulae, resection of the mandibular, mobilization of the genioglossus muscle, latissimus dorsi flap, scapula transplant, osseous reconstruction and tumor stadium 3 or 4. Each item contributes one point to the study score. None of the patient achieved more than five points in the study score

The clinical parameters which showed significant influence on the composite primary measure in the univariate analysis are shown in Table 3. Out of these data we developed a score with one point per matching item (0–8 points). However none of the patients in our cohort met more than five scoring criteria. Six patients met five study score criteria (Fig. 4). Five of these patients met the primary study endpoint. However a scoring threshold of five would have resulted in 16 undetected patients who required secondary tracheostomy or early reintubation. None of the possible thresholds of our study score predicted the right number of patients requiring secondary tracheostomy or early reintubation.

**Table 3:** Identified risk factors for secondary tracheostomy or early reintubation (univariate analysis)

Criteria		Rate of composite primary measure	Significance
Length of operation exceeding 75% quartile	yes	27.1%	*p* < 0.001
	no	2.9%	
pars alveolaris mandibulae	yes	14.7%	*p* = 0.049
	no	6.3%	
resection of mandibula	yes	12.9%	*p* = 0.037
	no	4.6%	
mobilization m.genioglossus	yes	62.5%	p < 0.001
	no	6.8%	
latissimus dorsi flap	yes	40%	p < 0.001
	no	5.3%	
scapula transplant	yes	50%	*p* = 0.001
	no	7.2%	
osseous reconstruction plate	yes	16.5%	*p* = 0.007
	no	5.2%	
Tumor stadium 3 or 4	yes	17.5%	*p* = 0.017
	no	5.8%

Validation of the items of the study with a multivariate regression analysis revealed only length of operation as risk factor with significant influence on the primary endpoint. The multivariate analysis is shown in Additional file 1.

## Discussion

The main result of our feasibility and safety study to evaluate a routine delayed extubation protocol after MOCS is that common clinical scoring systems fail to predict the need for tracheostomy. They indicated too many and the wrong patients for tracheostomy in our cohort. Usage of these scoring systems would have led to a much higher tracheostomy rate. Our data indicate that extubation in the ICU instead of routine tracheostomy or scoring based tracheostomy decision is superior in terms of safety, mortality or morbidity.

As we observed neither severe airway obstruction related morbidity or mortality nor any emergency surgical airway procedure, we assume that delayed routine extubation in the ICU is a safe and feasible method to avoid tracheostomy after MOCS. Avoidance of tracheostomy is not a therapeutic goal of its own. Tracheostomy related morbidity could harm patients severely and result in prolonged ICU and hospital stays, repeated operative tracheostomy revisions, reduced functional rehabilitation, tracheal operations due to strictures and lifelong impairment of the upper airway. Tracheostomy complication rates are reported to be 47% with tracheostomy caused 30 day hospital readmission rate of 13% [19]. Tracheostomy attributed mortality is reported 0.62% for surgical and 0.67% for dilative tracheostomies [4] and 1% in a smaller cohort [19]. The frequently documented comorbidities in the group of patients with oral cancer like chronical alcohol and nicotine abuse, local radiation therapy and infections of the upper and lower airway might contribute to an even higher rate of tracheostomy related morbidity in this patient group.

Abandonment of liberally performing tracheostomy after MOCS is posing a challenge to the ICU team. As long as ICU is staffed following recommendations by the European Society for Intensive Care Medicine the risk for controlled extubation of difficult airway patients in the ICU is minimal however [20]. We report two airway related cardiac arrests. However, with advanced airway management equipment and experienced staff available day and night, these situations were resolvable and did not lead to hypoxic deficit. Furthermore our results show that even in patients requiring early reintubation a secondary tracheostomy is not mandatory. Our study has certain limitations and lacks of a primary tracheostomy control group to compare primary and secondary outcome variables. However, the absence of severe complications in our study made us conclude that routine extubation in the ICU after MOCS is not inferior in terms of safety. One might think that delayed extubation in the ICU leads to a prolonged sedation and mechanical ventilation time. However, sedation was immediately stopped in favour of an analgetic opiod based regimen after ICU admission of the patients and 75% of the patients were extubated within 17 h after ICU admission following current guidelines for sedation and analgesia in the ICU [21, 22]. To our knowledge only three groups report a comparison of a primary tracheostomy group compared with a delayed extubation group in smaller cohorts. Two reports demonstrated that an overnight intubation protocol results in a shorter ICU and hospital stay in patients after MOCS [23, 24]. Meerwein and colleagues report a trend to earlier resumption of oral feeding and a decreased length of hospitalization in the no tracheostomy group [25]. We conclude that extubation in the ICU does not lead to prolonged ventilation times and does not increase ICU stay.

Previously published data of clinical scoring systems which aim to predict the need for primary tracheostomy were compared with the data of our patients. Objective of this evaluation was to assess whether the scores were suitable to predict the need for tracheostomy. None of the evaluated scores [13–16] was able to achieve a satisfying positive predictive value which means that none of the scores was able to identify the patients who required further airway management in the ICU. Furthermore the use of the Gupta [14] and the Cameron [16] scores would have resulted in a much higher rate of tracheostomies. The overall rate of tracheostomies suggested by the Kruse [15] and the Kim [13] score were nearly as high as observed in our cohort. However the true positive rates of both scores are low and the false positive rates are high which means that the scores didn’t identify the right patients. The ubiquitous application of the evaluated scores must be taken into question. To our knowledge none of the scores was ever evaluated with a high accuracy in other than their own cohorts [26]. The interinstitutional differences regarding difficult airway management, personal experiences and the degree of surgical resection to prevent airway obstruction might be too large to develop a universally reliable score.

Unfortunately, the score we developed out of our data was not able to predict the need for early reintubation or secondary tracheostomy properly. An underlying cutoff of 5 points would have resulted in 6 patients suggested for tracheostomy of which one patient showed no need for tracheostomy. A lower cutoff of 4 suggested 23 patients for tracheostomy of which 16 patients showed no need for secondary tracheostomy or reintubation. The poor reliability of our own score might be attributed to the small number of index patients. As all evaluated scores including our study score failed to identify the right patients requiring reintubation or secondary tracheostomy we are convinced that our approach of routine extubation in the ICU after MOCS is appropriate when performed by experienced staff. This approach could prevent patients from unnecessary potentially harmful tracheostomies.

## Conclusions

Availability of difficult airway management equipment like staged extubation kits, airway exchange catheters, videolaryngoscopic and fiberoptic devices as well as trained and experienced staff at day and night, delayed routine extubation in the ICU of patients who underwent MOCS is a safe and feasible method. This approach leads to a much lower incidence of tracheostomies than the use of formerly published clinical scoring systems.

## Additional files

Additional file 1: Additional statistical analysis (shows univariate and multivariate Analysis of potential risk factors for tracheostomy). (DOCX 20 kb)

Additional file 2: Details of the evaluated clinical scoring systems (shows the parameters of each of the evaluated scoring systems). (DOCX 17 kb)

Additional file 3: STROBE- Checklist (shows the filled in STROBE checklist). (DOCX 20 kb)

## Abbreviations

ASA: American Society of Anesthesiologists
BMI: Body mass index
ICU: Intensive care unit
MOCS: Major oral cancer surgery
OR: Operation room
PACS: Picture archiving and communication system
