# Supplementary material for: Protocol based evaluation for feasibility of extubation compared to clinical scoring systems after major oral cancer surgery safely reduces the need for tracheostomy: a retrospective cohort study
Source: BMC Anesthesiol. 2018 Apr 20;18:43. doi: 10.1186/s12871-018-0506-8 (PMC5910593; doi:10.1186/s12871-018-0506-8)
Supplement: Supplementary file 1 — Additional statistical analysis (shows univariate and multivariate Analysis of potential risk factors for tracheostomy). (DOCX 20 kb) [file 12871_2018_506_MOESM1_ESM.docx]

**Common clinical scoring systems fail to prevent tracheostomy in patients after major oral cancer surgery.**

Axel Schmutz^1^ M.D., Rolf Dieterich^1^, Johannes Kalbhenn^1^ M.D., Pit Voss² M.D., Torsten Loop^1^ M.D., Sebastian Heinrich^1^ M.D.*

* Sebastian Heinrich is the corresponding author

axel.schmutz@uniklinik-freiburg.de
johannes.kalbhenn@uniklinik-freiburg.de
rolf.dieterich@uniklinik-freiburg.de
pit.voss@uniklinik-freiburg.de
torsten.loop@uniklinik-freiburg.de
sebastian.heinrich@uniklinik-freiburg.de

^1^ Department of Anaesthesiology and Critical Care Medicine

Medical Center - University of Freiburg, Faculty of Medicine

Hugstetter Strasse 55

79106 Freiburg, Germany

Phone: +49 761 270 23060

Fax: +49 761 270 23960

^2^ Department of Oral and Maxillofacial Surgery & Regional Plastic Surgery

Medical Center - University of Freiburg, Faculty of Medicine

Hugstetter Strasse 55

79106 Freiburg, Germany

Phone: +49 761 270 49160

Fax: +49 761 270 48770

**Additional file 1:**

**Additional statistical analysis**

1. **Univariate Analysis to identify risk factors for secondary tracheostomy or early reintubation**

| Criteria |  | rate of primary endpoint | Significance |
| --- | --- | --- | --- |
| age >65 | yes | 9.1% | p=1.0 |
|  | no | 8.9% |  |
| tumor relapse | yes | 12.0% | p=0.239 |
|  | no | 7.3% |  |
| neoadjuvant chemotherapy | yes | 0% | p=1.0 |
|  | no | 9.2% |  |
| neoadjuvant radiotherapy | yes | 0% | p=0.606 |
|  | no | 9.4% |  |
| **Length of operation exceeding 75% quartile** | yes | 27.1% | p<0.001 |
|  | no | 2.9% |  |
| buccal tumor localization | yes | 11.8% | p=0.520 |
|  | no | 8.5% |  |
| tumor infiltration of maxilla | yes | 10% | p=0.697 |
|  | no | 8.9% |  |
| tumor site mandible angle | yes | 12.9% | p=0.495 |
|  | no | 8.4% |  |
| tumor site mandible | yes | 12.1% | p=0.108 |
|  | no | 5.5% |  |
| **tumor site pars alveolaris mandibulae** | yes | 14.7% | p=0.049 |
|  | no | 6.3% |  |
| tumor site anterior lingua | yes | 4.5% | p=0.204 |
|  | no | 10.8% |  |
| tumor site floor of the mouth | yes | 10.9% | p=0.490 |
|  | no | 7.6% |  |
| tumor site soft palate | yes | 7.7% | p=1.0 |
|  | no | 9% |  |
| tumor site hard palate | yes | 0% | p=1.0 |
|  | no | 10% |  |
| anterior palate arch | yes | 12.5% | p=0.534 |
|  | no | 8.8% |  |
| tumor site posterior palate arch | yes | 16.7% | p=0.435 |
|  | no | 8.8% |  |
| osteoradionecrosis | yes | 20% | p=0.057 |
|  | no | 7.7% |  |
| repetitive operation | yes | 12.5% | p<0.305 |
|  | no | 7.7% |  |
| **resection of mandibula** | yes | 12.9% | p=0.037 |
|  | no | 4.6% |  |
| resection of maxilla | yes | 11.1% | p=0.668 |
|  | no | 8.8% |  |
| resection of tongue | yes | 3.1% | p=0.071 |
|  | no | 11.2% |  |
| buccal resection, cheek resection | yes | 6.7% | p=1.0 |
|  | no | 9.3% |  |
| floor of the mouth resection | yes | 13% | p=0.102 |
|  | no | 6.4% |  |
| tonsillectomy | yes | 14.3% | p=0.487 |
|  | no | 8.8% |  |
| palate resection | yes | 6.7% | p=1.0 |
|  | no | 9.2% |  |
| mobilization m.geiohyoideus | yes | 33% | p=0.253 |
|  | no | 8.9% |  |
| **mobilization m.genioglossus** | yes | 62.5% | p<0.001 |
|  | no | 6.8% |  |
| mobilization m.mylohyoideus | yes | 8.3% | p=1.0 |
|  | no | 9.4% |  |
| **latissimus dorsi flap** | yes | 40% | p<0.001 |
|  | no | 5.3% |  |
| radial forearm flap | yes | 10% | p=0.765 |
|  | no | 8.8% |  |
| fibula flap | yes | 27.3% | p=0.065 |
|  | no | 8.1% |  |
| **scapula transplant** | yes | 50% | p=0.001 |
|  | no | 7.2% |  |
| iliac flap + iliac crest | yes | 0% | p=1.0 |
|  | no | 9.2% |  |
| solitary iliac crest | yes | 25% | p=0.315 |
|  | no | 8.7% |  |
| vastus flap | yes | 0% | p=1.0 |
|  | no | 9.1% |  |
| minor reconstruction with artificial tissue | yes | 0% | p=0.029 |
|  | no | 10.8% |  |
| **osseous reconstruction plate** | yes | 16.5% | p=0.007 |
|  | no | 5.2% |  |
| unilateral neck dissection | yes | 5.7% | p=0.238 |
|  | no | 11% |  |
| bilateral neck dissection | yes | 12% | p=0.408 |
|  | no | 8.2% |  |
| **Tumor stadium 3 or 4** | yes | 17.5% | p=0.017 |
|  | no | 5.8% |  |
| BMI >35kg/m² | yes | 9.1% | p=1.0 |
|  | no | 9.0% |  |
| Mallampati Score 3 or 4 | yes | 10.8% | p=0.789 |
|  | no | 9.3% |  |
| ASA Score 3 or 4 | yes | 9.6% | p=0.815 |
|  | no | 8% |  |

1. **Multivariate Analysis for identified risk factors**

| **Pseudo-R-Quadrat** | |
| --- | --- |
| Cox und Snell | ,163 |
| Nagelkerke | ,358 |
| McFadden | ,293 |

| **Likelihood-Quotienten-Tests** | | | | |
| --- | --- | --- | --- | --- |
| Effekt | Kriterien für die Modellanpassung | Likelihood-Quotienten-Tests | | |
|  | -2 Log-Likelihood für reduziertes Modell | Chi-Quadrat | Freiheitsgrade | Signifikanz |
| Konstanter Term | 61,587^a^ | ,000 | 0 | . |
| OP Dauer länger als 75%Quartile | 70,497 | 8,910 | 1 | ,003 |
| Pars Alveolaris Mandibulae* | 61,914 | ,328 | 1 | ,567 |
| Resektion Mandibula* | 61,589 | ,002 | 1 | ,967 |
| Ablösen M. genioglossus* | 64,411 | 2,825 | 1 | ,093 |
| Latissimus dorsi lappen | 63,859 | 2,272 | 1 | ,132 |
| Scapulatransplantat* | 61,587 | ,000 | 1 | ,986 |
| Knöcherne Rekonstruktion* | 61,632 | ,045 | 1 | ,832 |
| T-Stadium 3 oder 4 | 61,670 | ,083 | 1 | ,774 |
