# Supplementary material for: Protocol based evaluation for feasibility of extubation compared to clinical scoring systems after major oral cancer surgery safely reduces the need for tracheostomy: a retrospective cohort study
Source: BMC Anesthesiol. 2018 Apr 20;18:43. doi: 10.1186/s12871-018-0506-8 (PMC5910593; doi:10.1186/s12871-018-0506-8)
Supplement: Supplementary file 2 — Details of the evaluated clinical scoring systems (shows the parameters of each of the evaluated scoring systems). (DOCX 17 kb) [file 12871_2018_506_MOESM2_ESM.docx]

**Common clinical scoring systems fail to prevent tracheostomy in patients after major oral cancer surgery.**

Axel Schmutz^1^ M.D., Rolf Dieterich^1^, Johannes Kalbhenn^1^ M.D., Pit Voss² M.D., Torsten Loop^1^ M.D., Sebastian Heinrich^1^ M.D.*

* Sebastian Heinrich is the corresponding author

axel.schmutz@uniklinik-freiburg.de
johannes.kalbhenn@uniklinik-freiburg.de
rolf.dieterich@uniklinik-freiburg.de
pit.voss@uniklinik-freiburg.de
torsten.loop@uniklinik-freiburg.de
sebastian.heinrich@uniklinik-freiburg.de

^1^ Department of Anaesthesiology and Critical Care Medicine

Medical Center - University of Freiburg, Faculty of Medicine

Hugstetter Strasse 55

79106 Freiburg, Germany

Phone: +49 761 270 23060

Fax: +49 761 270 23960

^2^ Department of Oral and Maxillofacial Surgery & Regional Plastic Surgery

Medical Center - University of Freiburg, Faculty of Medicine

Hugstetter Strasse 55

79106 Freiburg, Germany

Phone: +49 761 270 49160

Fax: +49 761 270 48770

**Additional file 2: Details of the evaluated clinical scoring systems**

**2.1 Cameron et al. (2009)**

| Scoring Factor |  |  | Score |
| --- | --- | --- | --- |
| Tumour Site | Cutaneous Mouth  Oropharynx | Buccal mucosae Maxilla Mandibular alveolus Anterior tongue Floor of mouth Soft palate Anterior pillar Tonsillar pillar Posterior tongue Hypopharynx | 0 0 0 1 1 2 3 3 4 4 4 |
| Mandibulectomy |  | No Yes | 0 1 |
| Bilateral Neck Dissection |  | No Yes | 0 3 |
| Reconstruction |  | None RFFF Other | 0 2 3 |
| Threshold score: 5 “ Patients at or above the threshold, are at an increased risk of upper airway compromise. An elective tracheostomy should be consid- ered by the managing clinicians.“ | | | |

**2.2 Kim et al. (2014)**

| Scoring factor | Subsection | Score |
| --- | --- | --- |
| TNM Stage | I II III IV | 0 1 2 3 |
| Reconstruction | No reconstruction  Soft tissue free flap Soft+hard tissue free flap | 0 1 2 |
| Chest PA | No pathological findings Pathological findings | 0 1 |
| Number of systemic diseases | None 1-2 ≥3 | 0 1 2 |
| Cutoff value: 5 ≤5: no recommendation for elective tracheostomy, <5: recommendation for elective tracheostomy | | |

**2.3 Kruse-Lösler et al. (2005)**

| Points | 0 | 1 | 2 | 3 | 4 | Value |
| --- | --- | --- | --- | --- | --- | --- |
| Tumour localization |  | Anterior second premolars | Posterior second premolars |  |  | 1-2 |
| Tumour size |  | T1 | T2 | T3 | T4 | 1-4 |
| Chest X-ray | Normal | Pathological findings |  |  |  | 0-1 |
| Multimorbidity | No | Yes |  |  |  | 0-1 |
| Alcohol consumption | No | <100g/day | >100g/day | Hard drinks |  | 0-3 |
| ≥7 tracheotomy is recommended 6 risk collective of uncertain prognosis 2-5 tracheotomy is not necessary | | | | | | |

**2.4 Gupta et al. (2016)**

|  | Score |
| --- | --- |
| Major criterion |  |
| Previously radiated in same region of surgery | 2 |
| Resection oft wo more sub-sites of oral cavity or oropharynx | 2 |
| Bilateral neck dissection | 2 |
| Extended hemi or central arch mandibulectomy | 2 |
| Bulky flap for reconstruction: latissimus dorsi, double skin island pectoralis major myocutaneous flap | 2 |
| Flap with a compressing element: intact mandibular rim, use of concomitant reconstruction plate | 2 |
| Minor criterion: |  |
| Age >65 years | 1 |
| Previously operated at the same site | 1 |
| Trismus (Inter-incisor distance <1cm) | 1 |
| Pathological CT chest findings (COPD, emphysema, etc.) | 1 |
| Total score ≤6: Suggestive of no need for a tracheostomy Total score ≥7: Indicative of a need for a tracheostomy | |
