# Supplementary material for: Protocol based evaluation for feasibility of extubation compared to clinical scoring systems after major oral cancer surgery safely reduces the need for tracheostomy: a retrospective cohort study
Source: BMC Anesthesiol. 2018 Apr 20;18:43. doi: 10.1186/s12871-018-0506-8 (PMC5910593; doi:10.1186/s12871-018-0506-8)
Supplement: Supplementary file 3 — STROBE- Checklist (shows the filled in STROBE checklist). (DOCX 20 kb) [file 12871_2018_506_MOESM3_ESM.docx]

**Common clinical scoring systems fail to prevent tracheostomy in patients after major oral cancer surgery.**

Axel Schmutz^1^ M.D., Rolf Dieterich^1^, Johannes Kalbhenn^1^ M.D., Pit Voss² M.D., Torsten Loop^1^ M.D., Sebastian Heinrich^1^ M.D.*

* Sebastian Heinrich is the corresponding author

axel.schmutz@uniklinik-freiburg.de
johannes.kalbhenn@uniklinik-freiburg.de
rolf.dieterich@uniklinik-freiburg.de
pit.voss@uniklinik-freiburg.de
torsten.loop@uniklinik-freiburg.de
sebastian.heinrich@uniklinik-freiburg.de

^1^ Department of Anaesthesiology and Critical Care Medicine

Medical Center - University of Freiburg, Faculty of Medicine

Hugstetter Strasse 55

79106 Freiburg, Germany

Phone: +49 761 270 23060

Fax: +49 761 270 23960

^2^ Department of Oral and Maxillofacial Surgery & Regional Plastic Surgery

Medical Center - University of Freiburg, Faculty of Medicine

Hugstetter Strasse 55

79106 Freiburg, Germany

Phone: +49 761 270 49160

Fax: +49 761 270 48770

**Additional file 3: STROBE- Checklist**

STROBE Statement—checklist of items that should be included in reports of observational studies

|  | Item No | Recommendation |
| --- | --- | --- |
| **Title and abstract** | 1 | (*a*) Indicate the study’s design with a commonly used term in the title or the abstract **[Within the title page 1 and method section of the abstract page 2]** |
|  |  | (*b*) Provide in the abstract an informative and balanced summary of what was done and what was found **[See results section of the abstract page 2]** |
| Introduction | | |
| Background/rationale | 2 | Explain the scientific background and rationale for the investigation being reported **[See introduction section page 4]** |
| Objectives | 3 | State specific objectives, including any prespecified hypotheses **[See page 4, end of introduction section]** |
| Methods | | |
| Study design | 4 | Present key elements of study design early in the paper **[See methods section page 5-6]** |
| Setting | 5 | Describe the setting, locations, and relevant dates, including periods of recruitment, exposure, follow-up, and data collection **[See methods section page 5]** |
| Participants | 6 | (*a*) *Cohort study*—Give the eligibility criteria, and the sources and methods of selection of participants. Describe methods of follow-up **[See methods section page 5-6]**  *Case-control study*—Give the eligibility criteria, and the sources and methods of case ascertainment and control selection. Give the rationale for the choice of cases and controls **[not applicable]**  *Cross-sectional study*—Give the eligibility criteria, and the sources and methods of selection of participants **[not applicable]** |
|  |  | (*b*) *Cohort study*—For matched studies, give matching criteria and number of exposed and unexposed **[not applicable, we have chosen an unmatched study design]**  *Case-control study*—For matched studies, give matching criteria and the number of controls per case **[not applicable]** |
| Variables | 7 | Clearly define all outcomes, exposures, predictors, potential confounders, and effect modifiers. Give diagnostic criteria, if applicable **[See methods section page 5-6]** |
| Data sources/ measurement | 8* | For each variable of interest, give sources of data and details of methods of assessment (measurement). Describe comparability of assessment methods if there is more than one group **[See methods section page 5-6]** |
| Bias | 9 | Describe any efforts to address potential sources of bias **[See methods section page 5-6]** |
| Study size | 10 | Explain how the study size was arrived at **[See page 5 method section]** |
| Quantitative variables | 11 | Explain how quantitative variables were handled in the analyses. If applicable, describe which groupings were chosen and why **[The statistical processing is described in the method section]** |
| Statistical methods | 12 | (*a*) Describe all statistical methods, including those used to control for confounding  **[The statistical processing is described in the method section]** |
|  |  | (*b*) Describe any methods used to examine subgroups and interactions |
|  |  | (*c*) Explain how missing data were addressed **[Where occurred, missing data is stated in the result table, primary and secondary measurements show no missing data ]** |
|  |  | (*d*) *Cohort study*—If applicable, explain how loss to follow-up was addressed **[not applicable - there is no follow up section in the study]**  *Case-control study*—If applicable, explain how matching of cases and controls was addressed **[not applicable]**  *Cross-sectional study*—If applicable, describe analytical methods taking account of sampling strategy **[not applicable]** |
|  |  | (*e*) Describe any sensitivity analyses **[not applicable]** |

| Results | | |
| --- | --- | --- |
| Participants | 13* | (a) Report numbers of individuals at each stage of study—eg numbers potentially eligible, examined for eligibility, confirmed eligible, included in the study, completing follow-up, and analysed **[See results section page 6; figure 2]** |
|  |  | (b) Give reasons for non-participation at each stage **[not applicable]** |
|  |  | (c) Consider use of a flow diagram **[See figure 2]** |
| Descriptive data | 14* | (a) Give characteristics of study participants (eg demographic, clinical, social) and information on exposures and potential confounders **[See table 1]** |
|  |  | (b) Indicate number of participants with missing data for each variable of interest **[Where occurred, missing data is stated in the results (table 1), primary and secondary measurements show no missing data ]** |
|  |  | (c) *Cohort study*—Summarise follow-up time (eg, average and total amount) addressed **[not applicable - there is no follow up section in the study]** |
| Outcome data | 15* | *Cohort study*—Report numbers of outcome events or summary measures over time **[See figure 2]** |
|  |  | *Case-control study—*Report numbers in each exposure category, or summary measures of exposure **[not applicable]** |
|  |  | *Cross-sectional study—*Report numbers of outcome events or summary measures **[not applicable]** |
| Main results | 16 | (*a*) Give unadjusted estimates and, if applicable, confounder-adjusted estimates and their precision (eg, 95% confidence interval). Make clear which confounders were adjusted for and why they were included **[there was no adjustment to confounding variables]** |
|  |  | (*b*) Report category boundaries when continuous variables were categorized **[A single continuous variable was categorized within the study: Mallampati Score 1&2 vs. 3&4]** |
|  |  | (*c*) If relevant, consider translating estimates of relative risk into absolute risk for a meaningful time period **[not applicable]** |
| Other analyses | 17 | Report other analyses done—eg analyses of subgroups and interactions, and sensitivity analyses **[The analysis of score sensitivities, positive predictive values and specificity is stated in the method and result section (table 2)]** |
| Discussion | | |
| Key results | 18 | Summarise key results with reference to study objectives **[See discussion section page 7]** |
| Limitations | 19 | Discuss limitations of the study, taking into account sources of potential bias or imprecision. Discuss both direction and magnitude of any potential bias **[See discussion section page 8]** |
| Interpretation | 20 | Give a cautious overall interpretation of results considering objectives, limitations, multiplicity of analyses, results from similar studies, and other relevant evidence **[See discussion section page 8-9]** |
| Generalisability | 21 | Discuss the generalisability (external validity) of the study results **[See discussion section page 9]** |
| Other information | | |
| Funding | 22 | Give the source of funding and the role of the funders for the present study and, if applicable, for the original study on which the present article is based **[there is no conflict of interest declared]** |

*Give information separately for cases and controls in case-control studies and, if applicable, for exposed and unexposed groups in cohort and cross-sectional studies.

**Note:** An Explanation and Elaboration article discusses each checklist item and gives methodological background and published examples of transparent reporting. The STROBE checklist is best used in conjunction with this article (freely available on the Web sites of PLoS Medicine at http://www.plosmedicine.org/, Annals of Internal Medicine at http://www.annals.org/, and Epidemiology at http://www.epidem.com/). Information on the STROBE Initiative is available at www.strobe-statement.org.
